# Prevalence of Atrial Fibrillation and Long-Term Survival of Older Adults; Findings from the SNAC Study

**DOI:** 10.1177/23337214241304887

**Published:** 2024-12-03

**Authors:** Terese Lindberg, Johan Sanmartin Berglund, Anders Wimo, Chengxuan Qiu, Doris M. Bohman, Sölve Elmståhl

**Affiliations:** 1 Blekinge Institute of Technology, Karlskrona, Sweden; 2 Blekinge Center of Competence, Karlskrona, Sweden; 3 Lund University, Sweden; 4 Stockholm University, Sweden; 5 Karolinska Institutet, Stockholm, Sweden; 6 Uppsala University, Sjukhuset i Gävle, Sweden

**Keywords:** atrial fibrillation, long-term survival, older adults, prevalence, SNAC

## Abstract

**Objective:** This study examined the prevalence and long-term survival of atrial fibrillation (AF) in the older population. **Methods:** Data was recruited from the longitudinal SNAC study from baseline (2001–2004) for up to 10 years. **Results:** The population comprised 6,904 persons (59% women) (mean age 73.9 years). The prevalence of AF was 4.9% and increased with age. The hazard ratio (HR) for death in those with AF at baseline was 1.29 during the 10-year observation period. Cox regression analysis in persons with AF (*n* = 341) showed that men had a higher HR for death (1.57). CHA2DS2-VASc scores were significantly associated with death within 10 years (HR 1.29/score). Any form of anticoagulant use was reported in 146 (42.8%) and was significantly associated with survival (*p* = .031). **Conclusions:** The prevalence of AF in the general population was almost 5%, and it shortened life expectancy by nearly 2.4 years over a 10-year period. Despite the proven efficacy of OAC therapies, our results demonstrate that AF continues to be associated with increased mortality, especially among men, and that many older people are at high risk of developing a stroke because they do not receive appropriate anticoagulant therapy. These results emphasize the need for improved preventive and therapeutic modalities.

## Introduction

Atrial fibrillation (AF) is a complex disease that is highly prevalent in older adults (Andersson et al., 2012) and in persons with comorbidities (Zoni-Berisso et al., 2014). AF remains a significant public health challenge worldwide, with substantial variation at regional and national levels. There is an urgent need to increase public awareness about AF risk factors and to bring about cost-effective interventions for AF in order to reduce its future burden (Xu et al., 2022). Previous studies suggest that gender differences exist in mortality rates associated with AF and that AF diminishes the female survival advantage (Chugh et al., 2014). Furthermore, AF is strongly associated with increased mortality (Odutayo et al., 2016). Data from the Framingham Study indicate that AF exerts a significant impact on the risk of stroke independent of the often-associated cardiovascular abnormalities (Schnabel et al., 2015). In patients with AF, the average annual stroke rate is about 1.5% (Kirchhof et al., 2013) and as a guideline for the management of AF, the Congestive heart failure or left ventricular dysfunction, Hypertension, Age ≥75 (doubled), Diabetes, Stroke (doubled), Vascular disease, Age 65 to 74, and Sex category (female) (CHA_2_DS_2_-VASc) can be used. It provides a risk calculation for stroke and helps guide the decision (Camm et al., 2012) to prescribe oral anticoagulation and new oral anticoagulants (Ruff et al., 2014).

The European Society of Cardiology guidelines (ESC) recently recommended opportunistic screening for AF in men and women 65 years and older who seek medical care (Hindricks et al., 2021; Van Gelder et al., 2024). However, the National Board of Health and Welfare (The National Board of Health and Welfare, 2016) in Sweden suggested that more research was needed in order to recommend systematic screening. Understanding the pattern and trend of the atrial fibrillation (AF) burden is essential for developing effective preventive strategies (Xu et al., 2022). Both large-scale and smaller streamlined screening studies will provide information to inform policymakers in deciding whether national AF screening programs would be clinically and cost-effective, possible to implement at scale, and how acceptable this would be to patients (Jones et al., 2020). There is still a lack of evidence for the sufficiency of screening programs and the impact of early detection of AF on its clinical outcomes (Aras et al., 2022). AF appears to be common in the general population and has significant health implications. Still, only a few long-term prospective studies using random samples from the general population and detailed data at a national level are lacking. This calls for additional longitudinal studies in order to prevent complications and comorbidities that represent significant risk factors for AF. This study aims to determine the prevalence and long-term survival of AF among older adults in the general population in Sweden.

## Methods

**Figure 1.**
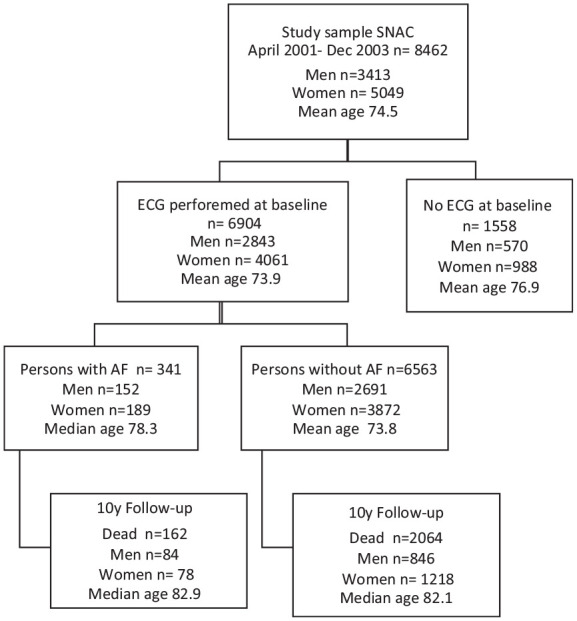
Flowchart of study population. *Note*. AF = atrial fibrillation; ECG = electrocardiogram; y = years.

This study included participants from the Swedish National Study on Aging and Care (SNAC). The SNAC study is an ongoing national, longitudinal, multidisciplinary study of aging, health, and care among individuals 60 and older. Data is continuously collected at four research centers in Sweden: Malmö, Karlskrona, Stockholm, and Nordanstig. The characteristics of the total SNAC sample are similar to those in the general population of older people in Sweden, and the study design is described in detail elsewhere (Lagergren et al., 2004). Briefly, the baseline examination of SNAC participants was conducted from 2001 to 2004. The SNAC sample was randomly recruited from 9 age cohorts (aged 60, 66, 72, 78, 81, 84, 87, 90, and 93 years). Of the total sample of individuals drawn from the municipality registers, 8,462 participated in the baseline examination (2001–2004). Most participants (81.2%) were examined at geriatric outpatient clinics or a research center. A total of 1,558 participants (18.4%) were examined in their homes or nursing homes; therefore, no electrocardiogram [ECG] examinations were performed. These persons were consequently excluded from the present study (Figure 1).

All data in the present study were collected from physical examinations and questionnaires.

To be included in the study, each person had to have undergone a 12s resting 12 lead ECG during the physical examination. Diagnosis of AF was based on the resting ECG on one occasion. Baseline sociodemographic (age, gender), clinical data, and stroke risk factors based on the CHA_2_DS_2_-VASc score, medication use (defined as acetylsalicylic acid [ASA] and warfarin and analyzed separately) were self-reported and collected during the examinations. The vital status of all participants was determined from data in the national death registry on October 30, 2015.

Descriptive statistics and graphical methods were used to characterize the data. The Chi-square test for significance was used to test for differences between persons with and without AF, age, and gender. For the survival analysis, Kaplan Meier and univariate Cox proportional hazards models were used to investigate the relationships in subjects with evidence of AF at the baseline examination between gender, CHA_2_DS_2_-VASc score, and age separately. Except for models exploring the effect of sex on survival, men and women were analyzed separately. Analyses were carried out using SPSS, version 23.0 (IBM Corporation, Armonk, NY, USA). The probability values (*p* values) are given in statically significant results. *p*-Values of less than .05 were considered significant. Missing data are noted by registering the number of participants (n) in all analyses.

## Results

At baseline, the study sample included 8,462 participants (3,413 men and 5,049 women), with a mean age of 74.5 years. The follow-up period began at the time of the individual’s baseline examination and ended on October 30, 2015, or at the time of death. A total of 2,929 (34%) persons died during the observation period. Of the baseline participants, 1,558 (18.5%) persons did not receive a resting ECG and were excluded from further analyses. The remaining study population (*n* = 6,904) had a mean age of 73.9 years, and 2,226 (32.2%) of these individuals died during the observation period. Demographic characteristics and a summary of the medical history of included groups and internal dropouts are compared in Table 1.

**Table 1.** Medical History of Those Receiving or Not Receiving an Electrocardiogram (ECG) and in Subjects With and Without Atrial Fibrillation.

Medical history	Total (*n* = 8,462) %	ECG examination			Atrial fibrillation		
		No (*n* = 1,558) %	Yes (*n* = 6,904) %	*p*-Value	No (*n* = 6,563) %	Yes (*n* = 341) %	*p*-Value
Death in 10 years	34.6	65.2	19.4	.000	17.7	49.0	.000
Smoker/former smoker	49.1	36.7	50.8	.050	51.1	47.0	.005
Diabetes	7.9	10.9	7.2	.315	7.1	10.1	.033
Stroke/TIA	8.4	15.7	6.8	.000	6.4	14.2	.001
Vascular disease	34.3	43.2	32.2	.836	30.2	70.6	.000
Congestive heart failure	5.5	7.8	5.0	.000	4.5	14.7	.000
Hypertension	28.3	30.7	27.8	.314	32.7	24.4	.041

*Note*. TIA = transient ischemic attack.

The study sample was in this study, divided into four age cohorts: (60, 66), (72, 78), (81, 84, 87) and 90 years and older. In the group with AF (*n* = 341, 4.9%), the median age was 78.3 years (range, 60–96 years) and included 152 men and 189 women (sex difference was not significant; Table 2). The prevalence of AF increased in the age cohorts and was 3% in those aged 60 and 66 years, 5.5% in those aged 72 and 78 years, and 7.4% in those aged 81, 84, and 87 years. The prevalence decreased to 5.7% in the oldest group, aged more than 90 years. A total of 162 persons (47.5%) with AF died during the observation period. A total of 164 (47%) persons reported that they smoked or were former smokers at the baseline examination (nonsignificant for survival). Furthermore, 49 persons (14.2%) (nonsignificant for sex difference) of those with AF had experienced a stroke.

**Table 2.** Age-Specific Prevalence of Atrial Fibrillation (AF) by Sex (*n* = 6,904).

Age group (years)	No. of subjects	No. of subjects with AF	Prevalence, % [95% CI]
*Men*			
60, 66	1,345	35	2.60 [1.75, 3.45]
72, 78	696	47	6.67 [4.76, 8.44]
81, 84, 87	629	55	8.74 [6.54, 10.95]
90+	173	15	8.67 [4.48, 12.86]
Total	2,843	152	5.35 [4.52, 6.17]
*Women*			
60, 66	1,487	49	3.30 [2.39, 4.21]
72, 78	999	47	4.70 [3.39, 6.01]
81, 84, 87	1,058	69	6.52 [5.03, 8.01]
90+	517	24	
Total	4,061	189	4.65 [4.01, 5.30]

*Note*. CI = confidence interval.

**Figure 2.**
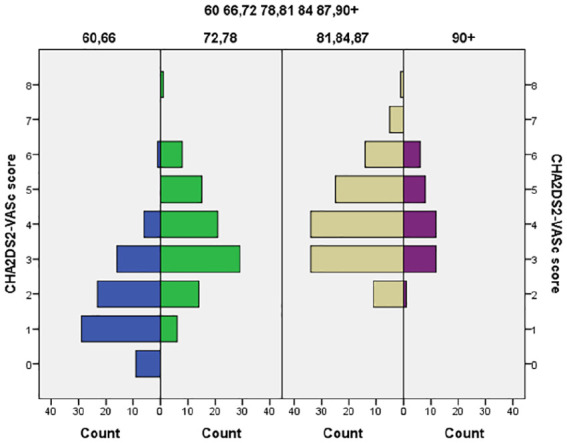
Distribution of CHA_2_DS_2_-VASc score in persons with AF (*n* = 341). *Note*. CHA_2_DS_2_-VASc = Congestive heart failure, Hypertension, Age ≥ 75 (doubled), Diabetes, Stroke (doubled), Vascular disease, Age 65 to 74, and Sex category (female).

The mean CHA_2_DS_2_-VASc score among persons with AF (*n* = 341) was 3.41 (95% CI: [3.24, 3.48]; range 1–8) compared with 2.62 (95% CI: [2.59, 2.66]) in those without AF. The mean CHA_2_DS_2_-VASc score for men with AF was 2.89 (95% CI: [2.68, 3.10]), and among women, it was 4.21 (95% CI: [4.0, 4.42]); this difference was significant (*p* < .01). The distribution of CHA_2_DS_2_-VASc score among age cohorts with AF is shown in Figure 2.

Sex differences and the distribution of CHA_2_DS_2_-VASc scores among persons with AF are shown in Table 3.

**Table 3.** Distribution of CHA_2_DS_2_-VASc Score in Persons with Atrial Fibrillation by Sex (*n* = 341).

CHA_2_DS_2_-VASc score	Total (*n* = 341)		Men (*n* = 152)		Women (*n* = 189)	
	No.	% [95% CI]	No.	% [95% CI]	No.	% [95% CI]
0	9	2.6 [0.91. 4.29]	9	5.6 [1.94. 9.26]	0	—
1	35	10.2 [6.99. 13.41]	18	11.8 [6.67. 16.93]	17	9.0 [4.92. 13.08]
2	49	14.4 [10.67. 18.13]	27	16.9 [10.94. 22.86]	22	11.6 [7.03. 16.17]
3	91	26.7 [22.0. 31.4]	55	36.1 [28.46. 43.74]	36	19.0 [13.41. 24.59]
4	73	21.4 [17.05. 25.75]	28	18.4 [12.24. 24.56]	45	24.0 [17.91. 30.09]
5	48	14.0 [10.32. 17.68]	12	7.9 [3.61. 12.19]	36	19.0 [13.41. 24.59]
6	29	8.5 [5.54. 11.46]	3	1.8 [0.0. 3.91]	26	13.8 [8.88. 18.72]
7	5	1.4 [0.15. 2.65]	0	—	5	2.7 [0.39. 5.01]
8	2	0.5 [0. 1.25]	0	—	2	0.5 [0.0. 1.51]
9	0	—	0	—	0	—

*Note*. CHA_2_DS_2_-VASc = Congestive heart failure, Hypertension, Age ≥75 (doubled), Diabetes, Stroke (doubled), Vascular disease, Age 65 to 74, and Sex category (female); CI, confidence interval.

### Survival Analyses

**Figure 3.**
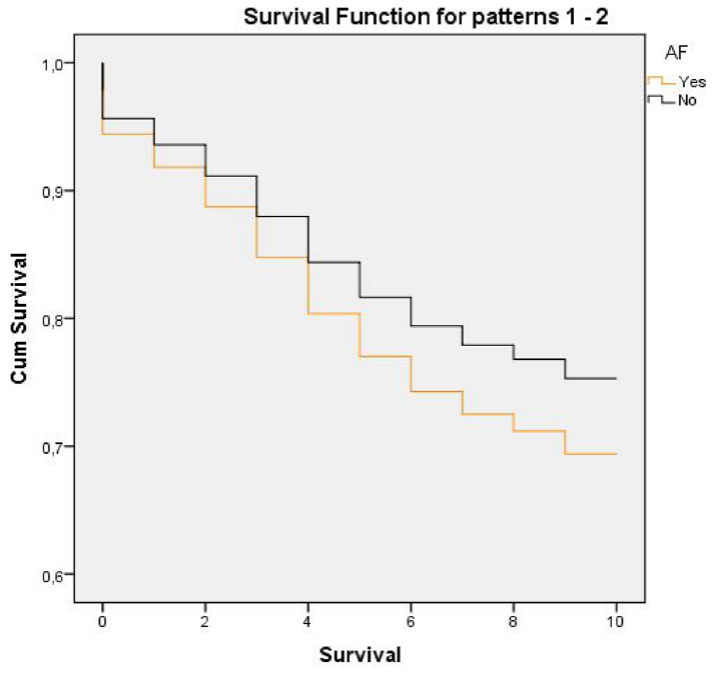
Ten-year survival in subjects with and without atrial fibrillation (age and gender adjusted). *Note*. Cum = cumulative.

The overall estimated (age-adjusted) 10-year survival of the study population was 9.29 years (95% CI: [9.25, 9.33]). The survival time was 9.28 (95% CI: [9.22, 9.35]) years for men and 9.29 (95% CI: [9.24, 9.34]) years for women (no difference between genders). Among persons without AF, the mean overall estimated (age-adjusted) 10-year survival was 9.41 (95% CI: [9.37, 9.44]) years compared with 7.03 (95% CI: [6.65, 7.4]) years (*p* < .01) in persons with AF (*n* = 341). Cox regression analysis in the total sample (*n* = 6,904) between persons with and without AF (age and gender adjusted) showed that AF was significantly related to death (*p* < .01). Cox regression analysis between having AF or not gave a HR of 1.29 (95% CI: [1.10, 1.51]) (Figure 3).

**Figure 4.**
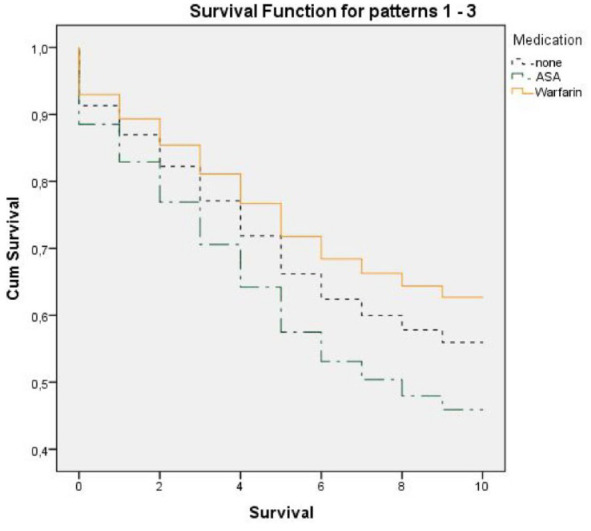
Comparison of 10-year survival in subjects with atrial fibrillation (*n* = 341) for persons treated with warfarin, ASA, or without anticoagulant therapy (CHA_2_DS_2_-VASc score adjusted). *Note*. CHA_2_DS_2_-VASc = Congestive heart failure, Hypertension, Age ≥ 75 (doubled), Diabetes, Stroke (doubled), Vascular disease, Age 65 to 74, and Sex category (female); Cum = cumulative.

Cox regression analysis between men and women (age adjusted) with AF (*n* = 341) gives a HR 1.57 (95% CI: [1.15, 2.13]) for men (*p* < .01). CHA_2_DS_2_-VASc score was significantly associated with an increased risk of death with a HR 1.29/score (95% CI: [1.10, 1.51]). An oral anticoagulant use was reported in 49 (14%) of 149 persons who received warfarin. Ninety-seven persons (28%) received ASA. Individuals with higher CHA_2_DS_2_-VASc scores had anticoagulants to a higher extent than those with a lower score. The use of anticoagulants (CHA_2_DS_2_-VASc score adjusted) in persons with AF was not significant (*p* = .439) for survival in Cox regression analysis, while the Cox regression analysis on warfarin and ASA separately showed that warfarin was significantly associated with survival (*p* = .031) (Figure 4).

## Discussion

This study investigated the prevalence of AF in the general population of older people in Sweden and their long-term survival. The prevalence of AF in our study was 4.9% and was strongly associated with increasing age, except for a decreasing prevalence in the oldest persons. This finding is probably caused by survival effects, leaving the healthiest to achieve the oldest ages. Additionally, the presence of AF might have been underestimated, particularly among the oldest old, in whom a higher number live in assisted care homes. Earlier results confirm that worldwide incidence and prevalence of AF show an increasing trend, which can be even sharper than expected in previous reports (Lippi et al., 2021). Reported prevalence rates among other studies differ widely, and the actual prevalence in the general population is uncertain because most published studies are based on data from registers or hospital populations, and they vary in age and setting (e.g., inpatient or outpatient cohorts, developed or developing countries, etc.). The results of such studies vary, as might be expected, and consequently highlight the fact that we need further research on AF in different settings. There are studies consistent with our results. In the Rotterdam study (Heeringa et al., 2006), the prevalence of AF increased with age from 0.7% in the age group 55 to 59 years to 17.8% in those aged 85 years and older. Compared to that study, our age-matched prevalence was significantly lower in the oldest age group. A lower prevalence in the oldest old was also seen in a report by SBU (2013) reporting on a general population in Sweden. They found a mean prevalence of AF of 2.9%, rising from 0.6% in the age group <60 years to 13.4% in those aged 80 to 89 years and decreasing to 9.0% in those aged 90 years and older. Also, Zoni-Berisso et al. (2014) reported prevalence figures similar to the present study in a European review. They reported a prevalence of AF of 0.12% to 0.16% in subjects younger than 49 years, 3.7% to 4.2% in those aged 60 to 70 years, and 10% to 17% in those aged 80 or older. The findings in the present study indicate no difference between the prevalence of AF in men and women, while Zoni-Berisso et al. (2014) reported that the prevalence of AF varies with sex. Furthermore, other studies showed that in both developed and developing countries, the age-adjusted prevalence and incidence of AF were lower in women (Chugh et al., 2014). AF was previously reported as associated with premature death (Benjamin et al., 1998), which was further confirmed in the present study. Almost half of the study population (49%) with AF died within 10 years. In persons without AF, the mean survival time was approximately 2.4 years longer compared to persons with AF, corresponding to a 72% lower risk of death after 10 years in those without AF. Other studies have reported that mortality associated with AF was higher in females (Chugh et al., 2014). The present study shows that mortality associated with AF was higher in men overall. The reason for this difference compared with previous studies is unclear but might depend on the study design. Our study constituted a randomized, population-based sample, including participants with and without a previously known AF diagnosis. Also, there were differences in comorbidities between men and women, and the frequency of warfarin treatment was lower in men than in women (nonsignificant). We did not look at the reasons for mortality. Nevertheless, since our sample is considered nationally representative of the older population in Sweden, we attribute it to high impact.

Despite improvement in the management of persons with AF, this arrhythmia remains one of the major causes of stroke, heart failure, sudden death, and cardiovascular morbidity in the world (Camm et al., 2012). In the present study, the mean CHA_2_DS_2_-VASc score was higher among persons with AF, indicating that comorbidity was higher in those with AF. Having more than one CHA_2_DS_2_-VASc score was shown to increase the risk of death during the 10-year observation period, with an average of 28.5% per score. Men with AF had a lower mean CHA_2_DS_2_-VASc score than women. Others reported that in men, AF is associated with a high incidence of ischemic heart disease, while heart failure is more common in women (Macfarlane et al., 2011). The average annual stroke rate is about 1.5%, and the annualized death rate is around 3% in patients with AF on anticoagulation treatment (Kirchhof et al., 2013). Earlier studies estimated anticoagulant use among persons with AF at between 40% and 60% (Oldgren et al., 2014). In the present study, the use of any form of anticoagulant was found in 42.8 % of those with AF, 97 persons (28%) received ASA, and only 14% received warfarin. Considering that those receiving aspirin and those left without anti-thrombotic agents have a higher risk of adverse clinical outcomes and mortality and that it may limit stroke prevention in AF (Bo et al., 2024; Guenoun et al., 2023) highlights the fact that several persons are living with a risk of stroke. Even though the prevention of stroke has had a marked improvement in the last 15 years with the availability of direct oral anticoagulants (Lip et al., 2023), treating older people with AF presents many challenges, including the balance between a risk for both stroke and bleeding (Foody, 2017). When prescribing OAC in the very old population, an individualized yet holistic approach accounting for comorbidities, comedications, altered physiological function, pharmacovigilance, frailty, compliance, and risk of fall is useful (Papazoglou et al., 2023). Taking this complexity into account could lead to not prescribing anticoagulation to older people; therefore, many patients still are inappropriately anticoagulated (Bo et al., 2024).

Individuals in the present study with higher CHA_2_DS_2_-VASc scores received anticoagulants to a higher extent than those with a lower score. The Cox regression analysis showed that overall anticoagulant use was not significant for survival, while warfarin itself was significantly associated with survival. This aligns with other reports that warfarin and antiplatelet agents reduce stroke by approximately 60% and 20%, respectively, in patients with AF (Hart et al., 2007).

Our study has limitations. The characteristics of the study sample, including gender, age, medical history, and death rate, differed between those included (those who received an ECG at the baseline examination) and those excluded from the analysis. People who were excluded had an overall higher rate of morbidity and mortality than those included in the analytical sample. This indicates that comparatively healthier individuals were included in the analysis and that the prevalence of AF was probably underrated. Another limitation is the lack of assessment of frailty, a variable that may be clinically and variably perceived as a determinant of outcome and condition prescription of anticoagulants (Diemberger et al., 2022; Savelieva et al., 2023). It turns out, for example, that anticoagulated patients with HF at baseline have higher rates of major bleeding and all-cause death (Schnabel et al., 2023). Considering that the impact of comorbidities affects the treatment of AF, optimized management and novel preventive strategies are required and feature the importance of taking care of comorbidities, as suggested by the ABC pathway, as it is associated with better outcomes (Ding et al., 2023; Patel et al., 2022).

This study uses the CHA2DS2VASc, but the most recent ESC AF Guidelines promote using CHA2DS2VA instead to assist in decisions on OCA therapy (Van Gelder et al., 2024). The reason ESC promotes CHA2DS2VA is that the sex component in the CHA2DS2-VASc score may no longer provide added predictive value in contemporary populations (Teppo et al., 2024). This study used a standard resting ECG for the diagnosis of AF. Previously reported observations (Kazemian et al., 2012) showed that up to 25% of cases of AF were undiagnosed by resting ECG since most cases of paroxysmal AF were not detected, which could lead to an underestimate of the prevalence of AF. It also highlights the fact that many more individuals live with a risk of developing stroke, considering that stroke rates are similar in both permanent and paroxysmal AF (Hohnloser et al., 2007).

Another thing to consider is that persons’ medical histories were self-reported, which could indicate that some of these conditions are underreported. An important strength of the present study is the population-based design, with a large nationally representative sample of older adults, including the oldest old. Research points out that large trials with clinical outcomes are needed to evaluate the potential benefits and harms of AF screening (Gladstone et al., 2021). Sweden has no national screening program as The National Board of Health and Welfare (The National Board of Health and Welfare, 2016) suggests that more research is needed to recommend systematic screening.

Our results are communicated through the SNAC, reporting to the government and the results to the general population to increase awareness about AF, allowing everyone to decide whether they would be interested in being tested and how frequently the testing should be repeated. The issue of searching for AF to detect asymptomatic AF is currently considered of great interest, and as a matter of fact, the more we look for AF, the more we find it, also using digital tools (Kalarus et al., 2023).

## Conclusion

The number of persons in the population with AF is increasing due to global aging. In this population-based study of older adults, the prevalence of AF was almost 5%. AF shortened life expectancy by nearly 2.4 years over 10 years. Despite the proven efficacy of current therapies such as anticoagulation, our results demonstrate that AF continues to be associated with increased mortality and that many older people have a high risk of developing a stroke since they do not receive appropriate anticoagulant therapy. Our findings emphasize the need for improved preventive and therapeutic modalities for this common arrhythmia. It also highlights the need for effective diagnostic tools and screening strategies in older persons to improve their long-term survival.
